# Mental Health in Children in the Context of COVID-19: Focus on Discharged Children

**DOI:** 10.3389/fpsyt.2021.759449

**Published:** 2021-11-11

**Authors:** Anyi Zhang, Le Shi, Wei Yan, Han Xiao, Yanping Bao, Zhe Wang, Jiahui Deng, Arun Ravindran, Kai Yuan, Hong Mei, Jie Shi, Zhisheng Liu, Jiajia Liu, Lin Lu

**Affiliations:** ^1^National Health Commission Key Laboratory of Mental Health (Peking University), National Clinical Research Center for Mental Disorders (Peking University Sixth Hospital), Peking University Institute of Mental Health, Peking University Sixth Hospital, Peking University, Beijing, China; ^2^Institute of Maternal and Child Health, Tongji Medical College, Wuhan Children's Hospital, Huazhong University of Science and Technology, Wuhan, China; ^3^Beijing Key Laboratory on Drug Dependence Research, National Institute on Drug Dependence, Peking University, Beijing, China; ^4^Department of Psychiatry, University of Toronto, Toronto, ON, Canada; ^5^Department of Neurology, Tongji Medical College, Wuhan Children's Hospital, Huazhong University of Science and Technology, Wuhan, China; ^6^School of Nursing, Peking University, Beijing, China; ^7^Peking-Tsinghua Center for Life Sciences and IDG/McGovern Institute for Brain Research at Peking University, Peking University, Beijing, China

**Keywords:** children, adolescent, mental health, COVID-19, PTSD

## Abstract

**Introduction:** To date, the mental health consequences of children hospitalized with COVID-19 remain unclear. We aimed to assess mental health status in children in the context of COVID-19, with a focus on discharged children.

**Methods:** We recruited discharged children who recovered from COVID-19 and healthy controls between July and September 2020 in Wuhan Children's Hospital. Post-traumatic stress disorder (PTSD), anxiety, depression, and sleep problems were assessed in these children using questionnaires. Univariable and multivariable logistic and linear regressions were conducted to identify risk factors.

**Results:** Totally, there were 152 children (61 discharged children and 91 healthy controls) aged 7–18 years old in our study. An increasing trend in the prevalence of PTSD, anxiety, and depression was observed in the discharged children compared with healthy controls (PTSD: 8.20 vs. 2.20%, anxiety: 22.95 vs. 13.19%; depression: 47.54 vs. 32.97%). Discharged children tended to report more depressive symptoms (β = 0.39) and less sleep problems (β = −0.37). Discharged children who lived in nuclear families and had longer hospital stays were more likely to report depression [odds ratio (OR) = 3.68 and 1.14, respectively]. Anxiety symptoms and the severity of sleep problems of discharged children were positively associated with caregivers' depression and PTSD symptoms (OR = 21.88 and 31.09, respectively).

**Conclusion:** In conclusion, PTSD, anxiety, and depression symptoms were common among recovered children 4 months after COVID-19 hospitalization. Children from nuclear family and those had longer hospital stays need special attention. In addition, parental mental health had a significant impact on their children's mental resilience and recovery.

## Introduction

By the end of May 2021, the coronavirus disease 2019 (COVID-19) pandemic afflicted more than 170 million people worldwide (1). The outbreak of COVID-19 has caused various mental health problems in the public and vulnerable populations, including healthcare workers, elderly, pregnant women, and children (2–8). Although children have a lower infection rate and relatively mild clinical symptoms, those with COVID-19 infection are considered more susceptible to mental health problems compared with unaffected children (9). Separation from caregivers and more mental health problems among caregivers that are caused by COVID-19 or close contact with confirmed cases can push affected children into a state of crisis and might increase their risk of psychiatric disorders (10). Additional stressors among children with COVID-19 may include hospital quarantine for prolonged periods of time, fear of the disease, being infected again, daily treatment, and the immunological response to infection (10).

Among the broader population, social distancing measures and school closures can also impact children's mental health. In a previous review, anxiety and depression were reported as the most prevalent psychological symptoms in children and adolescents during the COVID-19 pandemic (11). For example, a study in Wuhan, Hubei province, China, reported that depressive and anxiety symptoms affected 22.6 and 18.9% of primary students, respectively (12). In another study conducted in high school students during COVID-19 pandemic, 85.5% presented symptoms of post-traumatic stress disorder (PTSD), and post-pandemic PTSD in general population was 22.6% (13, 14). However, there were also positive impacts of the pandemic on children, as the school closure might protect children from school bullying or drug abuse (15). To date, most studies focused on the general population or healthy children, and evidence on children recovered from COVID-19 was scarce. Few studies evaluated the prevalence of psychological symptoms including anxiety, depression, and PTSD in children who were afflicted by COVID-19 and discharged from the hospital. Such studies are needed to determine appropriate support and resources. The present study (1) evaluated mental health status in children who had COVID-19 and were discharged from the hospital compared with healthy children who were quarantined at home and (2) explored risk factors that were associated with mental health problems in discharged children.

## Materials and Methods

### Study Design and Participants

This study had a case control design. Participants were recruited from a cohort of discharged pediatric patients who were hospitalized and treated for COVID-19 in Wuhan Children's Hospital between January and March 2020, and family member being infected was not a part of selection criteria. The diagnosis of COVID-19 was based on guidelines from the National Health Commission of the People's Republic of China and Society of Pediatrics of China (16). The physical and mental health status of these children were assessed from July to September 2020 after the lockdown for COVID-19 (i.e., 4 months after hospital discharge). Given the different measurements that are applied for children of different ages, only data on ≥7-year-old children were analyzed in the present study. We divided the participants into two age groups: school-aged children (7–12 years old) who were mostly in primary school and adolescents (13–18 years old) who were mostly in junior and senior high school. During the COVID-19 pandemic in 2020 in Wuhan, there were 363 children with COVID-19 infection hospitalized and treated in the Wuhan Children's Hospital. We recruited those participants through telephone, and 243 children excluded as they were not approached, 55 children were excluded as they were younger than 7 years old, 4 were excluded because of the incomplete questionnaire. So, there were 61 children left in the patient group. All participants in the COVID-19 patient group were diagnosed with asymptomatic or mild COVID-19. None of the patients had hypoxemia, were admitted to an intensive care unit, or used mechanical ventilation. In our study, children with asymptomatic or mild COVID-19 were accompanied by an uninfected family member. Children in the healthy control group were recruited from a birth cohort in Wuhan Children's Hospital, and their physical and mental health assessment was conducted during the same period as the COVID-19 patient group. Because of China's lockdown strategy, children in the healthy control group were quarantined at home during the COVID-19 pandemic.

### Ethics

The present study was reviewed and approved by the Ethics Committee of Wuhan Children's Hospital and complied with the Declaration of Helsinki. All of the children and their caregivers provided written informed consent.

### Measures

#### Socio-Demographic Characteristics

The children's socio-demographic characteristics were collected using the parent-reported questionnaire, including gender, age, ethnic, family structure, number of siblings, parents' age, household income, previous mental and physical health status, having family infected COVID-19 or not and family disease history.

#### PTSD

PTSD symptoms were assessed using the Child PTSD Symptom Scale (CPSS), which comprised 17 questions that assessed three key symptoms of PTSD: re-experiencing, avoidance, and arousal. Each item was rated on a 4-point Likert-type scale (17). Individuals who met the following criteria were identified as having PTSD: (1) at least one severity score > 2 among the five re-experiencing symptoms, (2) at least three severity scores > 2 among the seven avoidance symptoms, and (3) at least two severity scores > 2 among the five arousal symptoms.

#### Anxiety

The Screen for Child Anxiety Related Emotional Disorders (SCARED) was used to evaluate anxiety symptoms in children, which is a self-report questionnaire that consists of 41 items (18). All items were answered on a 3-point Likert-type scale. A total score ≥ 23 indicated significant symptoms of anxiety.

#### Depression

We used the 10-item Children's Depression Inventory-Short version (CDI-S) to assess depression status. A total score ≥ 3 indicated the likely presence of depression (19).

#### Sleep Problems

Sleep problems were assessed using the 26-item Sleep Disturbance Scale for Children (SDSC). Scores > 39 indicated sleep problems (20).

#### Post-traumatic Growth

This was assessed using 8 questions extracted from the 10-item Post-traumatic Growth Inventory–Short Form (PTGI-SF). Considering the different cultural backgrounds between China and Western countries, most Chinese people reported no religious beliefs (21), and this might lead to a lower score in the spiritual change subscale, so we deleted two items about spiritual change. In the present study, the Cronbach's α of the 8-item PTGI-SF was 0.91, which demonstrated a high internal consistency. Answers to the remaining 8-items were scored on a 4-point scale, ranging from 0 (not at all) to 3 (very much). A higher score indicated better PTG and greater positive change.

#### Trauma-Related Characteristics

We used the revised Brief Trauma Questionnaire (BTQ) to assess the retrospective history of exposure to traumatic events in children (22). The BTQ contains 12 types of traumatic events that may have occurred to respondents or family members. Answers were dichotomous (yes or no). The total score ranged from 0 to 12. Higher scores indicated more exposure to traumatic events before the COVID-19 pandemic.

#### Resilience

Resilience in children and adolescents was measured by the 10-item Connor–Davidson Resilience Scale (CD-RISC-10), extracted from the original 25-item CD-RISC (23). Higher scores indicated higher resilience capacity.

#### Perceived Social Support

Perceived social support was assessed using the 12-item Multidimensional Scale of Perceived Social Support (MSPSS), which has been well-validated in many countries and widely used in children and adolescents (24, 25). Higher scores indicated a higher level of perceived social support.

We also evaluated the mental health status of caregivers, the details of which are presented in the Supplementary Material.

### Statistical Analysis

Descriptive statistics were used to present the demographic characteristics of the COVID-19 patients and healthy controls. Demographic characteristics were compared using the χ^2^ test for categorical variables and Kruskal-Wallis test for continuous variables. The prevalence of PTSD, anxiety, depression, and sleep problems in children, stratified by demographic factors, are presented, and compared using the χ^2^ test. The total scores of questionnaires that assessed psychological conditions in children and caregivers were non-normally distributed. Therefore, these scores are presented as medians and interquartile ranges (IQRs) and were compared using the Kruskal-Wallis test. We also explored the likely contributors to psychiatric symptoms in children using univariate and multivariate regression models. The potential factors were initially identified using univariate linear and logistic regression models, the details of which are presented in the Supplementary Material. Factors were then identified using multivariate logistic and linear regression analyses with forward stepwise variable selection based on the Wald statistic (variables with *p* < 0.1) for entry into the final adjusted models. Odds ratios (ORs) and 95% confidence intervals (CIs) were determined for the logistic regression analyses, and the beta-coefficient (β) and 95% CI are presented for the linear regression analyses. Because of the non-normal distribution of total scores of the questionnaires, square root transformations were conducted for the linear regression models. The estimation of collinearity among parameters was calculated by the variance inflation factor (VIF) for the linear regression models. Variables with VIF <5 were entered into the final model (26).

The same analysis strategy was used for children who had recovered from COVID-19. All analyses were performed using Statistical Package for the Social Sciences (SPSS) 23.0 software. The level of significance was set at *p* < 0.05 (two-tailed).

## Results

### Demographic Characteristics

A total of 152 children and adolescents (61 discharged children and 91 healthy controls), 7–18 years old, were recruited in the present study. Among the discharged children, 13 (21.31%) were girls, which was lower than in healthy controls (45.06%; *p* = 0.010). There were 17 (27.87%) adolescents in the COVID-19 group, and 7 (7.69%) in the control group (*p* < 0.001). Among the discharged children, 75.41% (*n* = 46) reported having family with confirmed COVID-19. Among the healthy controls, 5.49% (*n* = 5) reported having family with confirmed COVID-19 (*p* < 0.001). The household income was significantly lower in discharged children compared with healthy controls (*p* < 0.01). Other demographic characteristics are shown in Table 1.

**Table 1:** Description and comparison of demographic characteristics (*n* = 152).

**Item**	**Total (*n* =152)**	**Discharged children (*n* = 61)**	**Healthy controls (*n* = 91)**	***p***
Girls [*n* (%)]	54 (35.53%)	13 (21.31%)	41 (45.06%)	**0.010**
**Age group [*****n*** **(%)]**				
School-aged children (7–12 years old)	120 (78.95%)	39 (63.93%)	81 (89.01%)	** <0.001**
Adolescent (13–18 years old)	24 (15.79%)	17 (27.87%)	7 (7.69%)	
Missing	8 (5.26%)	5 (8.20%)	3 (3.30%)	
Han ethnicity [*n* (%)]	141 (92.76%)	57 (93.44%)	84 (92.31%)	0.293
Nuclear family [*n* (%)]	65 (42.76%)	27 (44.26%)	38 (41.76%)	0.758
Being an only child [*n* (%)]	93 (61.18%)	39 (63.93%)	54 (59.34%)	0.569
Mother's age (years) (median, IQR)	38.00, 5.00	39.00, 6.50	37.00, 4.00	** <0.001**
Father's age (years) (median, IQR)	40.00, 6.00	42.00, 6.50	39.00, 5.00	** <0.001**
**Household income^a^** **[*****n*** **(%)]**				
**Before the pandemic**				
<5,000	10 (6.58%)	8 (13.11%)^*^	2 (2.20%)^*^	**0.010**
5,000–8,000	21 (13.82%)	13 (21.31%)^*^	8 (8.79%)^*^	
8,000–12,000	37 (24.34%)	15 (24.59%)	22 (24.18%)	
12,000–18,000	36 (23.68%)	10 (16.39%)	26 (28.57%)	
>18,000	34 (22.37%)	7 (11.48%)^*^	27 (29.67%)^*^	
Missing	14 (9.21%)	8 (13.11%)	6 (6.59%)	
**After the pandemic**				
<5,000	22 (14.47%)	18 (29.51%)^*^	4 (4.40%)^*^	** <0.001**
5,000–8,000	18 (11.84%)	11 (18.03%)	7 (7.69%)	
8,000–12,000	35 (23.03%)	10 (16.39%)	25 (27.47%)	
12,000–18,000	30 (19.74%)	9 (14.75%)	21 (23.08%)	
>18,000	29 (19.08%)	4 (6.58%)^*^	25 (27.47%)^*^	
Missing	18 (11.84%)	9 (14.75%)	9 (9.89%)	
Having family with confirmed COVID-19 [*n* (%)]	51 (33.55%)	46 (75.41%)	5 (5.49%)	** <0.001**
Previous mental disorders [*n* (%)]	2 (1.31%)	1 (1.64%)	1 (1.10%)	0.843
Family history of mental disorders [*n* (%)]	2 (1.31%)	1 (1.64%)	1 (1.10%)	0.953
Previous physical diseases [*n* (%)]	39 (25.66%)	15 (24.59%)	24 (26.37%)	0.247

*COVID-19, coronavirus disease 2019; IQR, interquartile range; SD, standard deviation*.

a *Household income in Chinese yuan per month as of August 31, 2020, 1 Chinese yuan = USD$0.14*.

* *Significant difference between COVID-19 group and healthy control group*.

*The significant p-values (p <0.05) are presented in bold*.

### Mental Health Status in Children With Different Epidemiological Characteristics

An increase trend in the prevalence of PTSD, anxiety, and depression was found in discharged children who recovered from COVID-19 compared with healthy controls (PTSD: 8.20 vs. 2.20%, p = 0.084; anxiety: 22.95 vs. 13.19%, p = 0.12; depression: 47.54 vs. 32.97%, p = 0.082). Among the total subjects, the prevalence of PTSD was higher in children whose caregivers reported PTSD (20.00 vs. 3.79%, p = 0.022) and poor sleep quality (10.53 vs. 1.15%, p = 0.016). In addition, the prevalence of anxiety in children was significantly higher in those whose caregivers reported depression (34.38 vs. 13.16%, p = 0.006). Depression severity was significantly higher in discharged children compared with healthy controls (median, IQR: 2.00, 6.00, and 2.00, 2.00, respectively; p = 0.023). In the whole participants, boys (median, IQR: 28.50, 11.00, and 31.00, 10.00, respectively; p = 0.044), the infected children (median, IQR: 27.00, 11.00, and 31.00, 10.00, respectively; p = 0.020), and nuclear family (median, IQR: 28.00, 9.00, and 31.00, 12.00, respectively; p = 0.045) were associated with fewer severe sleep problems. Children's sleep problems significantly increased in those whose caregivers reported PTSD (60.00 vs. 14.39%, p <0.001) and depression (40.63 vs. 11.40%, p <0.001). Additional data are presented in Table 2.

**Table 2:** Prevalence and severity of psychiatric symptoms in children stratified by epidemiological characteristics (n = 152).

**Item**	**PTSD^a^**		**Anxiety^b^**		**Depression^c^**		**Sleep problems^d^**	
	**Prevalence^e^ n (%)**	**Severity^f^ median, IQR**	**Prevalence^e^ n (%)**	**Severity^f^ median, IQR**	**Prevalence^e^ n (%)**	**Severity^f^ median, IQR**	**Prevalence^e^ n (%)**	**Severity^f^ median, IQR**
Total	7 (4.61%)	4.00, 8.00	26 (17.11%)	9.00, 14.00	59 (38.82%)	2.00, 3.00	26 (17.11%)	29.00, 10.00
**Gender**								
Girl	3 (5.56%)	4.00, 7.00	7 (13.96%)	9.00, 14.00	21 (40.38%)	2.00, 3.00	10 (19.23%)	31.00, 10.00
Boy	4 (4.40%)	4.00, 9.00	17 (18.68%)	9.00, 15.00	37 (41.57%)	2.00, 4.00	15 (17.05)	28.50, 11.00^*^
**Age group**								
7–12 years old	6 (5.00%)	3.00, 9.00	21 (17.50%)	9.00, 15.00	45 (38.79%)	2.00, 15.00	21 (18.10%)	29.00, 9.00
13–18 years old	1 (4.17%)	4.50, 9.00	3 (12.50%)	10.50, 12.00	13 (54.17%)	2.00, 5.00	3 (13.04%)	30.50, 13.00
**COVID-19 diagnosis**								
No	2 (2.20%)	3.00, 9.00	12 (13.19%)	9.00, 13.00	30 (32.97%)	2.00, 2.00	17 (19.54%)	31.00, 10.00
Yes	5 (8.20%)	5.00, 9.00	14 (22.95%)	10.00, 16.00	29 (47.54%)	2.00, 6.00^*^	9 (15.00%)	27.00, 11.00^*^
**Only child**								
No	3 (5.66%)	3.00, 9.00	9 (16.98%)	2.00, 3.00	21 (40.38%)	2.00, 5.00	6 (11.32%)	28.50, 7.00
Yes	4 (4.30%)	4.00, 8.00	16 (17.20%)	9.00, 16.00	37 (41.11%)	2.00, 3.00	19 (21.59%)	31.00, 12.00
**Nuclear family**								
No	4 (5.33%)	5.00, 8.00	11 (14.67%)	9.00, 13.00	26 (36.11%)	2.00, 3.00	10 (16.12%)	31.00, 12.00
Yes	3 (4.62%)	3.00, 9.00	11 (16.92%)	9.00, 17.00	28 (43.75%)	2.00, 5.00	14 (19.18%)	28.00, 9.00^*^
**Caregiver PTSD symptoms**								
No	5 (3.79%)	3.00, 8.00	23 (17.42%)	9.00, 14.00	50 (39.06%)	2.00, 3.00	19 (14.39%)	29.00, 9.00
Yes	2 (20.00%)^*^	11.50, 17.00	3 (30.00%)	15.50, 32.00	4 (40.00%)	3.00, 9.00	6 (60.00%)^**^	40.00, 25.00^*^
**Caregiver anxiety symptoms**								
No	4 (3.51%)	4.00, 8.00	20 (17.54%)	9.00, 15.00	45 (40.91%)	2.00, 4.00	19 (16.67%)	29.00, 9.00
Yes	3 (9.68%)	2.00, 11.00	6 (19.35%)	11.50, 15.00	10 (32.26%)	2.00, 2.00	6 (19.35%)	31.00, 14.00
**Caregiver depression symptoms**								
No	5 (4.39%)	3.00, 8.00	15 (13.16%)	9.00, 11.00	43 (39.09%)	2.00, 3.00	13 (11.40%)	29.00, 8.00
Yes	2 (6.25%)	7.50, 10.00	11 (34.38%)^**^	11.00, 21.00	12 (37.50%)	2.00, 5.00	13 (40.63%)^**^	35.00, 16.00^**^
**Caregiver sleep quality**								
Good	1 (1.15%)	4.00, 7.00	16 (18.39%)	10.00, 13.00	33 (38.82%)	2.00, 3.00	16 (18.39%)	29.00, 9.00
Poor	6 (10.53%)^*^	3.00, 11.00	10 (17.54%)	9.00, 15.00	22 (40.00%)	2.00, 6.00	9 (15.79%)	31.00, 12.00

*COVID-19, coronavirus disease 2019; PTSD, post-traumatic stress disorder; IQR, interquartile range*.

a *PTSD symptoms were assessed by the Child PTSD Symptom Scale, with the following definition of PTSD: (1) at least one severity score > 2 among the five re-experiencing symptoms, (2) at least three severity scores > 2 among the seven avoidance symptoms, (3) at least two severity scores > 2 among the five arousal symptoms*.

b *Anxiety in children was assessed by the Screen for Child Anxiety Related Emotional Disorders. Scores > 23 were defined as having anxiety*.

c *Depression in children was assessed by the Children's Depression Inventory-Short version. Scores > 3 indicated the likely presence of depression*.

d *Sleep problems in children were assessed by the Sleep Disturbance Scale for Children. Scores > 39 indicated having sleep problems*.

e *Comparisons of the prevalence of psychological symptoms in different groups were conducted using the χ^2^ test*.

f *The severity of psychological symptoms refers to assessment scores. Comparisons of the severity of psychological symptoms in different groups were conducted using the Kruskal-Wallis test*.

* *p <0.05*,

** *p <0.01*.

### Factors Related to Mental Health Symptoms in Children

**Figure 1:**
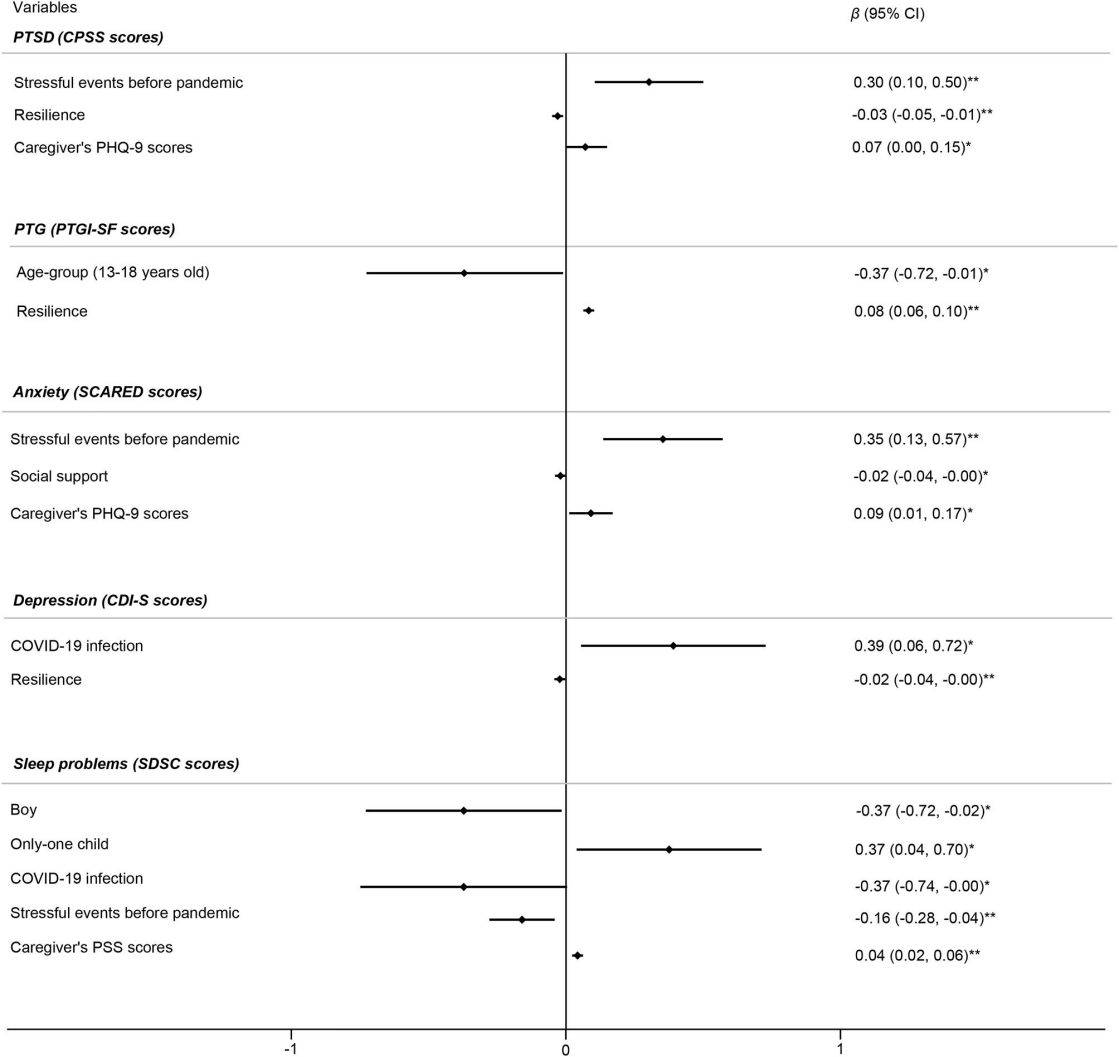
Factors associated with mental health symptoms in children (*n* = 152). Initial variables in the multivariate linear regression model for PTSD included gender, age group, COVID-19 diagnosis, stressful events before the pandemic, resilience, social support, and caregivers' PSS and PHQ-9 scores. Initial variables in the multivariate linear regression model for PTG included gender, age group, COVID-19 diagnosis, stressful events before the pandemic, resilience, social support, and caregivers' PSS and PHQ-9 scores. Initial variables in the multivariate linear regression model for anxiety included gender, age group, COVID-19 diagnosis, stressful events before the pandemic, resilience, social support, and caregivers' PHQ-9 scores. Initial variables in the multivariate linear regression model for depression included gender, age group, COVID-19 infection, nuclear family, stressful events before the pandemic, resilience, and social support. Initial variables in the multivariate linear regression model for sleep problems included gender, age group, COVID-19 diagnosis, being an only child, stressful events before the pandemic, and caregivers' PSS and PHQ-9 scores. Independent variables in the final model were determined using forward selection. *p < 0.05, **p < 0.01.

As shown in Figure 1, children who experienced more stressful events before the pandemic (β = 0.30, *p* = 0.004 for PTSD, β = 0.35, *p* = 0.002 for anxiety), children who had COVID-19 (β = 0.39, *p* = 0.019) and children with fewer siblings (β = 0.37, *p* = 0.035) reported worse mental health status. While, male gender (β = −0.37, *p* = 0.043), resilience (β = −0.03, *p* = 0.009 for PTSD, β = −0.02, *p* = 0.003 for depression) and social support (β = −0.02, *p* = 0.043) would protect children from developing psychological symptoms. In addition, caregiver's mental health status was closely related to children's mental health. When caregivers showed depression symptoms (β = 0.07, *p* = 0.048 for children's PTSD, β = 0.09, *p* = 0.025 for children's anxiety symptoms) or post-traumatic stress symptoms (β = 0.04, *p* = 0.001), children might report an increased risk for psychological symptoms. PTG was higher in children from primary school (β = −0.37, *p* = 0.043) and children who perceived more resilience (β = 0.08, *p* < 0.001). Interestingly, children who had COVID-19 (β = −0.37, *p* = 0.049) tended to have fewer sleep problems.

In the adjusted logistic regression model, the risk factors for mental health problems in the whole participants included stressful events before the pandemic (OR = 1.54, *p* = 0.025), elder age (OR = 1.17, *p* = 0.032), and the poor mental health status in caregivers. While, children's resilience (OR = 0.94, *p* = 0.003), perceived social support (OR = 0.97, *p* = 0.026), and higher household income (OR = 0.52, *p* = 0.008) might protect them against psychological symptoms. Moreover, children with COVID-19 infection had better sleep quality (OR = 0.24, *p* = 0.038). Details are presented in Table 3.

**Table 3:** Risk factors associated with psychiatric symptoms in children and adolescents (*n* = 152).

**Dependent variable**	**Independent variable in the final model**	**OR**	**95% CI**	***p***
PTSD	COVID-19 diagnosis	13.79	0.99, 192.61	0.051
	Caregiver PTSD	25.39	1.77, 363.62	0.017
	Caregiver poor sleep quality	13.97	0.99, 192.61	0.051
Anxiety	Stressful event before the pandemic	1.54	1.06, 2.23	0.025
	Social support	0.97	0.94, 1.00	0.026
	Caregiver depression	3.87	1.28, 11.70	0.016
Depression	Age group (13–18 years old)	1.17	1.01, 1.36	0.032
	Resilience	0.94	0.91, 0.98	0.003
Sleep problems	Higher household income after the pandemic	0.52	0.32, 0.84	0.008
	COVID-19 diagnosis	0.24	0.06, 0.93	0.038
	Caregiver PTSD	8.91	1.77, 44.89	0.008
	Caregiver depression	3.10	1.04, 9.25	0.043

*PTSD, post-traumatic stress disorder; OR, odds ratio; 95% CI, 95% confidence interval; COVID-19, coronavirus disease 2019*.

*Initial variables in the logistic regression model for PTSD included gender, age group (13–18 years old), COVID-19 diagnosis, social support, and caregivers' PTSD and poor sleep quality. Initial variables in the logistic regression model for anxiety included gender, age group (13–18 years old), COVID-19 diagnosis, stressful events experienced before the COVID-19 pandemic, resilience, and caregivers' depression. Initial variables in the logistic regression model for depression included gender, age group (13–18 years old), COVID-19 diagnosis, and resilience. Initial variables in the logistic regression model for sleep problems included gender, age group (13–18 years old), COVID-19 diagnosis, household income before and after the COVID-19 pandemic, and caregivers' PTSD and depression. Independent variables in the final model were determined using forward selection*.

### Factors Related to Mental Health Symptoms in Discharged Children

**Figure 2:**
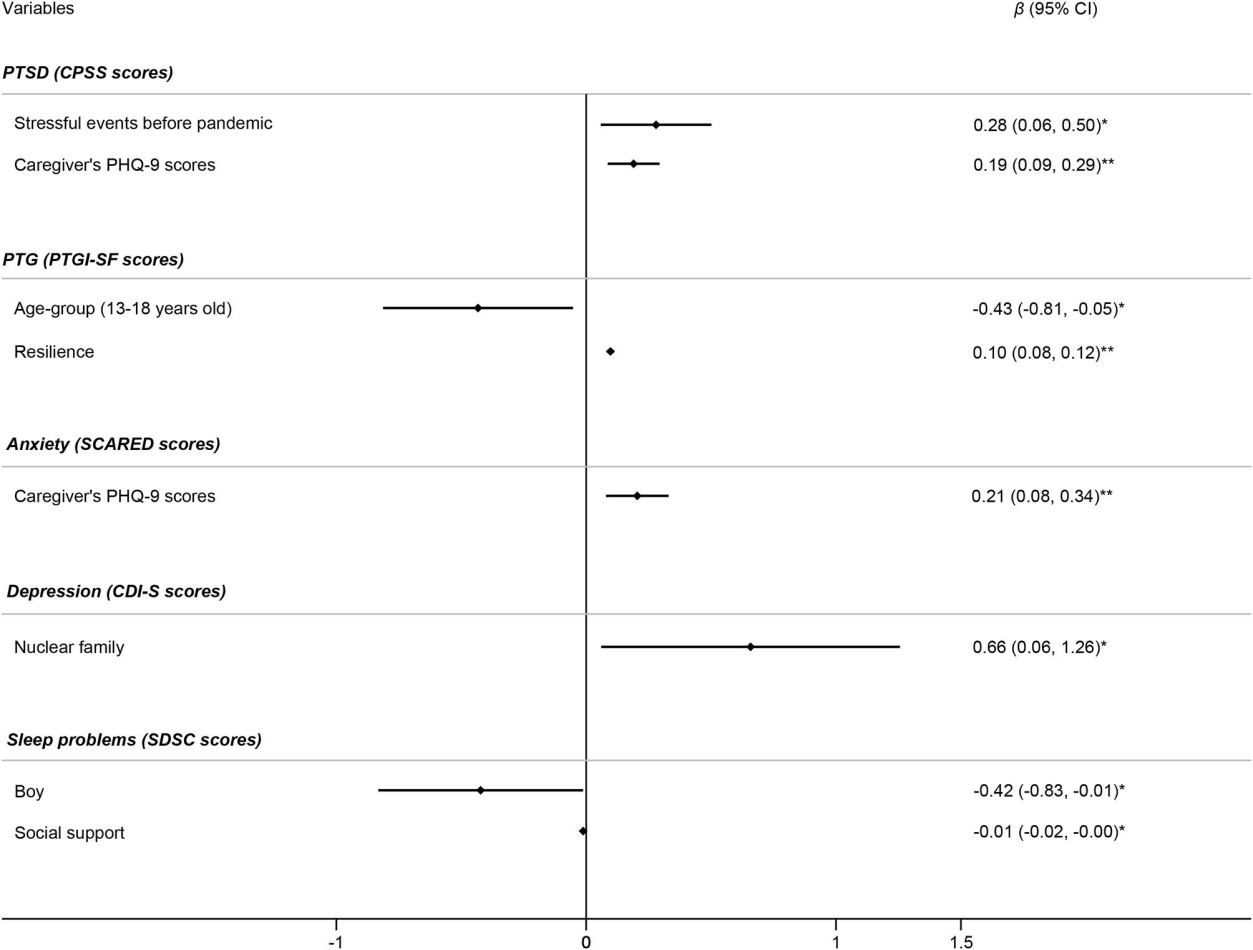
Factors associated with mental health symptoms in discharged children (*n* = 61). Initial variables in the multivariate linear regression model for PTSD included gender, age group, stressful events before the pandemic, and caregivers' GAD-7 and PHQ-9 scores. Initial variables in the multivariate linear regression model for PTG included gender, age group, resilience, social support, and caregivers' PHQ-9 and PSQI scores. Initial variables in the multivariate linear regression model for anxiety included gender, age group, and caregivers' PHQ-9 scores. Initial variables in the multivariate linear regression model for depression included gender, age group, being an only child, nuclear family, length of hospitalization, and social support. Initial variables in the multivariate linear regression model for sleep problems included gender, age group, being an only child, resilience, and social support. Independent variables in the final model were determined using forward selection. *p < 0.05, **p < 0.01.

In the discharged children, mental health problems tended to be severe or prevalent in those experienced more stressful events before pandemic (β = 0.28, *p* = 0.015). Caregivers' poor mental health status (e.g., depression symptoms) might in turn worsen the psychological symptoms in children. In accordance with the relationship in the total subjects, higher level of resilience might decrease the severity of mental health problems in discharged children. Boys (β = −0.42, *p* = 0.050) had fewer sleep problems (Figure 2).

In the logistic regression analysis, the risk factors for mental health problems in discharged children included caregivers' poor mental health, the nuclear family structure (OR = 3.68, *p* = 0.038) and the extended length of hospitalization (OR = 1.14, *p* = 0.045) (Table 4).

**Table 4:** Risk factors associated with psychiatric symptoms in discharged children (*n* = 61).

**Dependent variable**	**Independent variable in the final model**	**OR**	**95% CI**	***p***
Anxiety	Caregiver depression	21.88	3.15, 152.05	0.002
Depression	Nuclear family	3.68	1.07, 12.64	0.038
	Length of hospitalization	1.14	1.00, 1.30	0.045
Sleep problems	Caregiver PTSD	31.09	1.44, 671.10	0.028
	Higher household income after the pandemic	0.38	0.13, 1.07	0.068

*OR, odds ratio; 95% CI, 95% confidence interval*.

*Variables in the logistic regression model for anxiety included gender, age group (13–18 years old), stressful events experienced before the COVID-19 pandemic, social support, and caregivers' depression. Variables in the logistic regression model for depression included gender, age group (13–18 years old), nuclear family, and length of hospitalization. Variables in the logistic regression model for sleep problems included gender, age group (13–18 years old), higher household income before and after the COVID-19 pandemic, and caregivers' PTSD. Independent variables in the final model were determined using forward selection*.

## Discussion

To our knowledge, this was the first study to analyze long-term mental health status in children who recovered from COVID-19 and discharged from hospital. We found a significantly higher level of depression compared with healthy controls who had not contracted COVID-19 and an increased trend in prevalence of PTSD and anxiety. We also found that COVID-19 infection was significantly associated with depression symptoms and sleep problems in children. Moreover, caregivers' poor mental health status was an important risk factor for mental health problems in children, especially in children who recovered from COVID-19.

The prevalence of PTSD in the present study was 4.61% among all participants (8.20% in discharged children and 2.20% in healthy controls). Individuals, especially children and adolescents, are known to have a higher risk of developing PTSD following exposure to traumatic events (14). A previous study reported that nearly two-thirds of children and adolescents might be exposed to traumatic events, such as violence, accidents, or witnessing trauma events, and 4.7–7.8% of them develop PTSD afterward (27). In the present study, the risk of developing PTSD was higher in children who previously experienced more stressful events, which was consistent with a previous study (27). As the previous exposure to the traumatic events might increase or worsen the physical or emotional reaction to the stressors or trauma, prior traumatic events exposure might increase the vulnerability of PTSD in children and adolescent (27). Additionally, children's PTSD symptoms were associated with their caregivers' PTSD symptoms. A previous cohort study found that caregivers' exposure to traumatic events was associated with a higher prevalence of PTSD, which would contribute to a harsh parenting style and consequently increase the prevalence of mental health problems in children (28). Similar results were found among children who were hospitalized because of COVID-19. Our results highlight the significance of properly addressing children's psychological needs in a timely fashion both during and after pandemics.

Children's and adolescents' responses to traumatic events vary. Some might have symptoms of PTSD, but others might experience positive changes, including hope for new possibilities, positive changes in relationships with others, the growth of personal strength, changes in spirit, and a greater appreciation of life, which are important characteristics of PTG (29). A previous systematic review identified factors that were associated with PTG, child factors (age and gender), trauma factors (type of trauma and level of trauma exposure), psychological factors (resilience and coping), and family factors (parental psychological characteristics) (30). In the present study, children who were younger and had greater resilience tended to have higher PTG, regardless of whether they had COVID-19, which was consistent with a previous study that found that older age was associated with lower PTG (30).

The overall prevalence of anxiety was 17.11% (22.95% in discharged children and 13.19% in healthy controls). It was comparable to previous studies reported that the prevalence of anxiety in children during the COVID-19 pandemic was about 20% (12). At the individual level, treatment and quarantine in a hospital bring sudden changes to children's daily routines, and some of them are separated from social network, which can worsen existing symptoms or lead to new symptoms (10). Children's mental health is related to the family environment. This was observed in the present study, in which children's anxiety was positively related to their caregivers' depression symptoms. During the COVID-19 pandemic, many parents lost their jobs and household income, which might worsen caregivers' depression symptoms and further affect the mental health status of their children (31). Another important finding in the present study was the negative correlation between children's perceived social support and anxiety symptoms. Social support is a type of psychological adjustment that helps individuals cope with difficult times, including traumatic events (32). The protective function of social support was not observed in children with COVID-19 in the present study, which might be because of the relatively limited resources of social support that were available during hospitalization.

In the present study, nearly half of the children who recovered from COVID-19 had depression symptoms. A previous study in Wuhan, China, reported that the prevalence of depression was 26.5% among children who were quarantined at home during the pandemic, which was much lower than in the present study (12). During the COVID-19 outbreak, school closures, restricted outdoor activities, and lack of social interactions might have led to more mental health problems in children who were restricted at home (33). For inpatients with COVID-19, physical inactivity, possible invasive surgeries, a higher risk of repeated infection, and separation from social network were all additional stressors (10). Interestingly, children from nuclear families were more likely to develop depression after recovering from COVID-19, suggesting that increased attention should be paid to children with emotional problems in the nuclear families. In the present study, nearly half of the families were extended families that included more than one generation. Grandparents provide important support for maintaining normal family function and are essential resources of social support, which may promote family and personal resilience (34). Additionally, the dramatic increase in the prevalence of depression indicated that increases in stress and poor mental health status exist for a prolonged time in children and adolescents even after the pandemic, highlighting the necessity of early and continuous prevention and intervention strategies to address potential mental health symptoms both during and after pandemics.

In the present study, the overall prevalence of sleep problems was 17.11% (15.00% in discharged children and 19.54% in healthy controls). Girls, being an only child, and not having COVID-19 but being quarantined at home were associated with more sleep problems. Caregivers' depression symptom was another important risk factor for sleep problems in children. This is consistent with Caldwell et al., who found that mothers' life stress was significantly and negatively related to their children's sleep quality (35). Evidence of the effects of COVID-19 on children's sleep quality is mixed, possibly because of the different ages of children in different studies. Moore et al. found that COVID-19 lockdowns did not affect sleep in children (36). Di Giorgio et al. and Lecuelle et al. reported an increase in the prevalence of sleep problems after the COVID-19 lockdown (37, 38). Both of these studies were conducted in preschool children. In contrast, we focused on school aged children and found that discharged children had fewer sleep problems than children who were quarantined at home. This might be related to a regular schedule for the hospitalized children. These findings imply that interventions for sleep problems in children and adolescents are essential, especially for children who are quarantined at home.

The present study investigated the prevalence of mental health consequences and risk factors among Chinese pediatric patients who had COVID-19, were hospitalized, and then were discharged. The present study has limitations. First, the total sample size was relatively small, which might decrease statistical power. Only one in three of the children who were hospitalized during the pandemic agreed to follow-up physical and mental health examinations. Such a lack of willingness to participate in the present study might be because that the children or caregivers feared being stigmatized. Further studies with larger and representative sample sizes are needed. Second, mental health status of children and adolescents was a cross-sectional assessment. We are planning to conduct follow-up studies to analyze trajectories of mental health symptoms in pediatric patients. Third, children and adolescents in the control group were mainly enrolled from a birth cohort in Wuhan Children's Hospital, which began in 2012. As a result, the oldest children in this cohort were 8 years old. The median age of the healthy controls was younger than discharged children. Future studies are needed with matched samples.

In conclusion, mental health problems were common among children who had COVID-19, even 4 months after hospital discharge. Children who experienced more stressful events before the pandemic and whose caregivers reported more depressive symptoms were more likely to develop PTSD and anxiety. Discharged children, especially children in nuclear families, were more likely to suffer from depressive symptoms compared with healthy controls. Caregivers' PTSD symptoms were another risk factor for children's poor sleep after COVID-19 recovery. Interventions and strategies that focus on children's mental health outcomes should be developed and provided to children continuously even after pandemics. For children who have had COVID-19, specific attention should be paid to those from nuclear families, girls, and students from junior and senior high school. Protecting and maintaining the mental health of caregivers should also be considered an essential strategy to improve children's mental health after COVID-19 and quarantine.

## Data Availability Statement

The datasets presented in this article are not readily available because this dataset is shared and managed by the authors in Peking University Sixth Hospital and Wuhan Children's Hospital. It might not be opened without the agreement of data managers in Peking University Sixth Hospital and Wuhan Children's Hospital. Requests to access the datasets should be directed to Jiajia Liu, liujiajia_sdu@bjmu.edu.cn; Lin Lu, linlu@bjmu.edu.cn; Han Xiao, tjxiaohan1980@163.com; and Zhisheng Liu, liuzsc@126.com.

## Ethics Statement

The studies involving human participants were reviewed and approved by the Ethics Committee of Wuhan Children's Hospital. Written informed consent to participate in this study was provided by the participants' legal guardian/next of kin.

## Author Contributions

AZ, LS, WY, and HX wrote the initial draft of manuscript with contributions from JL and YB. AZ and JL developed the study design and analysis plan. AZ, HX, ZW, JL, and HM collected the questionnaires. AZ and JL conducted the data analyses with help from YB. LS, YB, JL, JD, and LL critically revised the manuscript. JS, ZL, JL, and LL supported and led the data collection and data analysis. All authors approved the results and final version.

## Funding

This study was supported by the Fundamental Research Funds of the Central Universities (no. 69004Y1230), National Natural Science Foundation of China (nos. 81761128036, 81821092, and 31900805), National Key Research and Development Program of China (no. 2019YFA0706200), PKU-Baidu Fund (2020BD011), and Peking University Medicine Fund of Fostering Young Scholars' Scientific and Technological Innovation and Fundamental Research Funds for Central Universities (no. BMU2020PYB013).

## Conflict of Interest

The authors declare that the research was conducted in the absence of any commercial or financial relationships that could be construed as a potential conflict of interest.

## Publisher's Note

All claims expressed in this article are solely those of the authors and do not necessarily represent those of their affiliated organizations, or those of the publisher, the editors and the reviewers. Any product that may be evaluated in this article, or claim that may be made by its manufacturer, is not guaranteed or endorsed by the publisher.
