# Supplementary material for: Mental Health in Children in the Context of COVID-19: Focus on Discharged Children
Source: Front Psychiatry. 2021 Nov 11;12:759449. doi: 10.3389/fpsyt.2021.759449 (PMC8631929; doi:10.3389/fpsyt.2021.759449)
Supplement: Supplementary file 1 [file Data_Sheet_1.docx]

**Mental health in children in the context of COVID-19: focus on discharged children**

Anyi Zhang,^1,†^ Le Shi, ^1,†^ Wei Yan,^1,†^ Han Xiao,^2,†^ Yanping Bao,^3^ Zhe Wang,^1^ Jiahui Deng,^1^ Arun Ravindran,^4^ Kai Yuan,^1^ Hong Mei,^2^ Jie Shi,^3^ Zhisheng Liu,^5,^ * Jiajia Liu,^6,^ * Lin Lu,^1, 7,^ *

**MATERIALS AND METHODS**

*Mental health status of caregivers*

*Posttraumatic stress disorder and posttraumatic growth*. Posttraumatic stress disorder (PTSD) symptoms in caregivers were measured using the 17-item PTSD Symptom Scale (PSS).(Mirzamani et al., 2007) Answers to each item were scored from 0 (not at all) to 4 (extremely). Caregivers with total scores > 37 had a higher risk of PTSD.(Coffey et al., 1998) Posttraumatic Growth was measured using the Posttraumatic Growth Inventory–Short Form (PTGI-SF), a 21-item scale that consists of a 6-point Likert-type scale from 0 (not at all) to 5 (to a great extent).(Tedeschi and Calhoun, 1996) Higher scores indicated better growth after a traumatic event.

*Anxiety and depression*. We used the 7-item Generalized Anxiety Disorder (GAD-7) and 9-item Patient Health Questionnaires (PHQ-9) to screen anxiety and depression symptoms in caregivers. A total score ≥ 5 indicated the presence of anxiety or depression.

*Sleep quality*. We evaluated sleep quality using the self-report Pittsburgh Sleep Quality Index (PSQI). A total score ≥ 11 indicated poor sleep quality. It is a reliable and standardized questionnaire that can distinguish between “good” and “poor” sleep quality.(Pilz et al., 2018)

*Statistical analysis*

We explored factors that may be potentially related to psychiatric symptoms in children using univariate linear and logistic regression analyses. Variables that were significantly different between groups were then entered into multivariate regression models using the forward stepwise (Wald) option to determine risk factors. Raw *β* coefficients, odds ratios (ORs), and 95% confidence intervals (CIs) were presented in this step.

Risk factors for mental health problems in discharged children were then analyzed using the same method. All of the analyses were performed using Statistical Package for the Social Sciences (SPSS) 23.0 software. The level of significance was set at *p* < 0.05 (two-tailed).

**Results**

As shown in **eTable 1**, COVID-19 diagnosis was related to a higher risk of depression (*β* = 0.39, *p* = 0.013) and a lower risk of sleep problems (*β* = -0.45, *p* = 0.025) in children. A significant correlation was found between children’s and caregivers’ mental health conditions. Children’s Child PTSD Symptom Scale (CPSS) scores (*β* = 0.09, *p* = 0.014), PTGI-SF scores (*β* = -0.07, *p* = 0.007), Screen for Child Anxiety Related Emotional Disorders (SCARED) scores (*β* = 0.10, *p* = 0.010), and Sleep Disturbance Scale for Children (SDSC) scores (*β* = 0.06, *p* = 0.042) were related to caregivers’ PHQ-9 scores. Measures of four types of children’s mental health conditions (CPSS scores, PTGI-SF scores, SCARED scores, and Children’s Depression Inventory-Short version [CDI-S] scores) were also related to social support and resilience.

According to the unadjusted logistic regression analysis (**eTable 2**), children perceived that social support (OR = 0.95, *p* = 0.011) would protect them from developing PTSD. Caregivers’ PTSD symptoms (OR = 6.35, *p* = 0.043) and poor sleep quality (OR = 10.12, *p* = 0.034) increased the risk of developing PTSD. Stressful events that children experienced before the pandemic (OR = 1.53, *p* = 0.005) and caregivers’ depression (OR = 3.46, *p* = 0.008) increased the prevalence of anxiety in children. Resilience (OR = 0.95, *p* = 0.030) and social support (OR = 0.96, *p* = 0.0048) were protective against anxiety. Depression in children was more prevalent in children with a lower level of resilience (OR = 0.95, *p* = 0.003). The prevalence of sleep problems increased in children whose caregivers reported PTSD (OR = 8.92, *p* = 0.002) and depression (OR = 5.32, *p* < 0.001). Socioeconomic factors, such as a higher household income, were protective against sleep problems in children.

The results of the univariate linear regression analysis for discharged children are shown in **eTable 3**. Caregiver’s PHQ-9 scores were positively related to three types of psychological conditions (PTSD, PTG, and anxiety) in their children. A positive correlation was found between caregivers’ GAD-7 scores and children’s CPSS scores (*β* = 0.17, *p* = 0.039). PTGI-SF scores were significantly lower in discharged children whose caregivers reported higher PSQI scores (*β* = -0.13, *p* = 0.011). Interestingly, the length of hospitalization (*β* = 0.06, *p* = 0.036) in discharged children was positively related to their depression symptoms. Children’s resilience and perceived social support protected against depression (*β* = -0.02, *p* = 0.017) and sleep problems (*β* = -0.01, *p* = 0.013). Other correlations are presented in **eTable3**.

The results of the univariate logistic regression analysis are shown in **eTable 4**. We found no potential risk factors for PTSD in discharged children. Stressful events that were experienced before the COVID-19 pandemic (OR = 1.52, *p* = 0.044) and caregivers’ depression (OR = 6.67, *p* = 0.006) increased the risk of anxiety in children. Perceived social support (OR = 0.96, *p* = 0.050) decreased the prevalence of anxiety. In accordance with the linear regression model, the length of hospitalization was positively related to depression symptoms in discharged children (OR = 1.13, *p* = 0.044). Interestingly, children from nuclear families had a higher prevalence of depression (OR = 3.99, *p* = 0.017) than children from extended families. A higher household income before (OR = 0.45, *p* = 0.040) and after (OR = 0.33, *p* = 0.039) the COVID-19 pandemic protected discharged children from sleep problems. PTSD symptoms (OR = 12.00, *p* = 0.014) in caregivers exacerbated sleep problems in children.

**eTable 1. The potential risk factors associated with children’s mental health problems (square root transformed) in univariate linear regression model (*n* = 152).**

| **Variable** | **PTSD (CPSS score)** | | **PTG (PTGI-SF score)** | | **Anxiety (SCARED score)** | | **Depression (CDI-S score)** | | **Sleep problems (SDSC score)** | |
| --- | --- | --- | --- | --- | --- | --- | --- | --- | --- | --- |
|  | ***β*** | **95% CI** | ***β*** | **95% CI** | ***β*** | **95% CI** | ***β*** | **95% CI** | ***β*** | **95% CI** |
| Gender |  |  |  |  |  |  |  |  |  |  |
| Girl | Ref | Ref | Ref | Ref | Ref | Ref | Ref | Ref | Ref | Ref |
| Boy | 0.06 | -0.45, 0.58 | 0.21 | -0.19, 0.60 | -0.28 | -0.86, 0.31 | 0.11 | -0.22, 0.44 | **-0.46*** | **-0.83, -0.10** |
| Age group |  |  |  |  |  |  |  |  |  |  |
| 7-12 years old | Ref | Ref | Ref | Ref | Ref | Ref | Ref | Ref | Ref | Ref |
| 13-18 years old | 0.13 | -0.56, 0.82 | 0.03 | -0.49, 0.55 | 0.19 | -0.57, 0.94 | 0.36 | -0.05, 0.78 | -0.05 | -0.54, 0.43 |
| Number of siblings |  |  |  |  |  |  |  |  |  |  |
| ≥ 1 | Ref | Ref | Ref | Ref | Ref | Ref | Ref | Ref | Ref | Ref |
| 0 | 0.20 | -0.32, 0.72 | 0.38 | -0.02, 0.77 | 0.17 | -0.41, 0.74 | -0.14 | -0.46, 0.18 | **0.41*** | **0.04, 0.78** |
| Family structure |  |  |  |  |  |  |  |  |  |  |
| Extended family | Ref | Ref | Ref | Ref | Ref | Ref | Ref | Ref | Ref | Ref |
| Nuclear family | -0.11 | -0.62, 0.39 | 0.08 | -0.30, 0.47 | -0.08 | -0.65, 0.49 | **0.34*** | **0.02, 0.67** | -0.28 | -0.65, 0.09 |
| COVID-19 diagnosis |  |  |  |  |  |  |  |  |  |  |
| No | Ref | Ref | Ref | Ref | Ref | Ref | Ref | Ref | Ref | Ref |
| Yes | 0.18 | -0.32, 0.68 | -0.13 | -0.51, 0.25 | 0.01 | -0.54 0.57 | **0.39*** | **0.08, 0.70** | **-0.45*** | **-0.85, -0.06** |
| Mother’s age | 0.02 | -0.04, 0.07 | -0.01 | -0.05, 0.04 | -0.02 | -0.08, 0.05 | 0.02 | -0.02, 0.05 | -0.03 | -0.07, 0.01 |
| Father’s age | 0.01 | -0.04, 0.07 | 0.02 | -0.02, 0.06 | 0.01 | -0.05, 0.07 | 0.02 | -0.01, 0.05 | -0.02 | -0.06, 0.02 |
| Higher household income before pandemic | -0.17 | -0.37, 0.04 | 0.09 | -0.07, 0.24 | -0.14 | -0.38, 0.09 | -0.06 | -0.19, 0.08 | 0.09 | -0.07, 0.24 |
| Higher household income after pandemic | -0.13 | -0.32, 0.06 | 0.07 | -0.07, 0.22 | -0.08 | -0.30, 0.13 | -0.07 | -0.19, 0.05 | 0.10 | -0.04, 0.24 |
| Stressful event before pandemic | **0.27**** | **0.11, 0.43** | **-0.13*** | **-0.25, -0.01** | **0.21*** | **0.03, 0.39** | **0.12*** | **0.02, 0.22** | **-0.14*** | **-0.27, -0.01** |
| Resilience | **-0.03**** | **-0.06, -0.01** | **0.08**** | **0.06, 0.09** | **-0.03*** | **-0.06, -0.01** | **-0.02**** | **-0.04, -0.01** | -0.00 | -0.02, 0.01 |
| Social support | **-0.03**** | **-0.04, -0.01** | **0.03**** | **0.02, 0.04** | **-0.03**** | **-0.05, -0.01** | **-0.02**** | **-0.03, -0.00** | -0.00 | -0.01, 0.01 |
| Caregivers’ PSS scores | **0.03*** | **0.00, 0.06** | **-0.03*** | **-0.05, -0.00** | 0.03 | -0.01, 0.06 | 0.00 | -0.02, 0.02 | **0.03*** | **0.01, 0.05** |
| Caregivers’ PTGI scores | -0.00 | -0.01, 0.01 | 0.01 | -0.00, 0.02 | -0.01 | -0.02, 0.00 | -0.00 | -0.01, 0.01 | 0.00 | -0.01, 0.01 |
| Caregivers’ GAD-7 scores | 0.03 | -0.05, 0.11 | -0.03 | -0.09, 0.04 | 0.04 | -0.05, 0.13 | -0.02 | -0.07, 0.04 | 0.00 | -0.06, 0.06 |
| Caregivers’ PHQ-9 scores | **0.09*** | **0.02, 0.16** | **-0.07**** | **-0.13, -0.02** | **0.10**** | **0.03, 0.18** | 0.02 | -0.03, 0.06 | **0.06*** | **0.00, 0.11** |
| Caregivers’ PSQI scores | 0.06 | -0.03, 0.14 | -0.06 | -0.12, 0.00 | -0.01 | -0.10, 0.08 | 0.03 | -0.03, 0.08 | 0.00 | -0.06, 0.06 |

COVID-19, coronavirus disease 2019; 95% CI, 95% confidence interval; PTSD, posttraumatic stress disorder; PTGI-SF, Posttraumatic Growth Inventory–Short Form; CDI-S, Children’s Depression Inventory-Short Version; SCARED, Screen for Child Anxiety Related Emotional Disorders; CPSS, Child PTSD Symptom Scale; SDSC, Sleep Disturbance Scale for Children; PSS, PTSD Symptom Scale; PTG-I, Posttraumatic Growth Inventory; PHQ-9, Patient Health Questionnaire, 9-item; GAD-7, Generalized Anxiety Disorder, 7-item; PSQI, Pittsburgh Sleep Quality Index; Ref, reference. **p* < 0.05, ***p* < 0.01.

**eTable 2. Potential risk factors associated with children’s psychiatric symptoms in univariate logistic regression model (*n* = 152)**

| **Variable** | **PTSD** | | | **Anxiety** | | | **Depression** | | | **Sleep problem** | | |
| --- | --- | --- | --- | --- | --- | --- | --- | --- | --- | --- | --- | --- |
|  | **OR** | **95% CI** | ***p*** | **OR** | **95% CI** | ***p*** | **OR** | **95% CI** | ***p*** | **OR** | **95% CI** | ***p*** |
| Gender |  |  |  |  |  |  |  |  |  |  |  |  |
| Girl | Ref | Ref | Ref | Ref | Ref | Ref | Ref | Ref | Ref | Ref | Ref | Ref |
| Boy | 0.78 | 0.17, 3.63 | 0.75 | 1.54 | 0.60, 4.00 | 0.37 | 1.05 | 0.52, 2.11 | 0.89 | 0.86 | 0.36, 2.09 | 0.74 |
| Age group |  |  |  |  |  |  |  |  |  |  |  |  |
| 7-12 years old | Ref | Ref | Ref | Ref | Ref | Ref | Ref | Ref | Ref | Ref | Ref | Ref |
| 13-18 years old | 0.83 | 0.10, 7.19 | 0.86 | 0.67 | 0.18, 2.47 | 0.55 | 1.87 | 0.77, 4.52 | 0.17 | 0.68 | 0.19, 2.50 | 0.56 |
| Number of siblings |  |  |  |  |  |  |  |  |  |  |  |  |
| ≥ 1 | Ref | Ref | Ref | Ref | Ref | Ref | Ref | Ref | Ref | Ref | Ref | Ref |
| 0 | 0.71 | 0.16, 3.48 | 0.71 | 1.01 | 0.41, 2.49 | 0.97 | 1.03 | 0.51, 2.07 | 0.93 | 2.16 | 0.80, 5.80 | 0.13 |
| Family structure |  |  |  |  |  |  |  |  |  |  |  |  |
| Extended family | Ref | Ref | Ref | Ref | Ref | Ref | Ref | Ref | Ref | Ref | Ref | Ref |
| Nuclear family | 0.86 | 0.19, 3.99 | 0.85 | 1.19 | 0.48, 2.95 | 0.72 | 1.38 | 0.69, 2.74 | 0.36 | 0.81 | 0.33, 1.98 | 0.65 |
| COVID-19 diagnosis |  |  |  |  |  |  |  |  |  |  |  |  |
| No | Ref | Ref | Ref | Ref | Ref | Ref | Ref | Ref | Ref | Ref | Ref | Ref |
| Yes | 3.97 | 0.75, 21.18 | 0.11 | 1.96 | 0.84, 4.60 | 0.12 | 1.81 | 0.92, 3.54 | 0.084 | 0.73 | 0.30, 1.76 | 0.48 |
| Mother’s age | 1.10 | 0.96, 1.26 | 0.16 | 0.99 | 0.90, 1.09 | 0.82 | 1.01 | 0.94, 1.08 | 0.85 | 0.98 | 0.88, 1.08 | 0.67 |
| Father’s age | 0.99 | 0.85, 1.16 | 0.91 | 1.02 | 0.94, 1.12 | 0.63 | 1.03 | 0.96, 1.10 | 0.42 | 1.01 | 0.93, 1.11 | 0.78 |
| Higher household income before pandemic | 0.73 | 0.40, 1.35 | 0.31 | 0.75 | 0.52, 1.09 | 0.13 | 0.80 | 0.60, 1.07 | 0.13 | **0.63** | **0.44, 0.91** | **0.013** |
| Higher household income after pandemic | 0.70 | 0.40, 1.24 | 0.22 | 0.86 | 0.61, 1.20 | 0.37 | 0.77 | 0.59, 1.00 | 0.053 | **0.67** | **0.48, 0.94** | **0.019** |
| Stressful event before pandemic | 1.35 | 0.96, 1.89 | 0.082 | **1.53** | **1.14, 2.05** | **0.005** | 1.12 | 0.89, 1.39 | 0.34 | 1.13 | 0.87, 1.46 | 0.36 |
| Resilience | 0.94 | 0.87, 1.01 | 0.11 | **0.95** | **0.91, 0.99** | **0.030** | **0.95** | **0.92, 0.98** | **0.003** | 0.98 | 0.94, 1.02 | 0.33 |
| Social support | **0.95** | **0.91, 0.99** | **0.011** | **0.96** | **0.93, 0.99** | **0.005** | 0.98 | 0.96, 1.00 | 0.085 | 0.99 | 0.96, 1.01 | 0.28 |
| Caregivers’ PTSD | **6.35** | **1.06, 37.98** | **0.043** | 2.03 | 0.49, 8.45 | 0.33 | 1.04 | 0.28, 3.87 | 0.95 | **8.92** | **2.30, 34.59** | **0.002** |
| Caregivers’ anxiety | 2.95 | 0.62, 13.93 | 0.17 | 1.13 | 0.41, 3.11 | 0.82 | 0.69 | 0.30, 1.60 | 0.39 | 1.20 | 0.43, 3.32 | 0.73 |
| Caregivers’ depression | 1.45 | 0.27, 7.87 | 0.66 | **3.46** | **1.39, 8.58** | **0.008** | 0.94 | 0.42, 2.11 | 0.87 | **5.32** | **2.14, 13.23** | **< 0.001** |
| Caregivers’ poor sleep quality | **10.12** | **1.18, 86.44** | **0.034** | 0.94 | 0.40, 2.26 | 0.90 | 1.05 | 0.53, 2.01 | 0.89 | 0.83 | 0.34, 2.04 | 0.69 |

PTSD, posttraumatic stress disorder; OR, odds ratio; COVID-19, coronavirus disease 2019; 95% CI, 95% confidence interval; Ref, reference.

**eTable 3. Potential risk factors associated with psychiatric symptoms (square root transformed) in discharged children in univariate** **linear regression model (*n* = 61).**

| **Variable** | **PTSD (CPSS score)** | | **PTG (PTGI-SF score)** | | **Anxiety (SCARED score)** | | **Depression (CDI-S score)** | | **Sleep problems (SDSC score)** | |
| --- | --- | --- | --- | --- | --- | --- | --- | --- | --- | --- |
|  | ***β*** | **95% CI** | ***β*** | **95% CI** | ***β*** | **95% CI** | ***β*** | **95% CI** | ***β*** | **95% CI** |
| Gender |  |  |  |  |  |  |  |  |  |  |
| Girl | Ref | Ref | Ref | Ref | Ref | Ref | Ref | Ref | Ref | Ref |
| Boy | -0.86 | -1.92, 0.20 | 0.38 | -0.41, 1.17 | -0.72 | -0.16, 0.53 | 0.17 | -0.54, 0.87 | -0.57 | -1.38, 0.25 |
| Age group |  |  |  |  |  |  |  |  |  |  |
| 7-12 years old | Ref | Ref | Ref | Ref | Ref | Ref | Ref | Ref | Ref | Ref |
| 13-18 years old | -0.14 | -0.19, 0.91 | 0.29 | -0.44, 1.02 | 0.24 | -0.91, 1.39 | 0.30 | -0.34, 0.93 | 0.15 | -0.59, 0.89 |
| Number of siblings |  |  |  |  |  |  |  |  |  |  |
| ≥ 1 | Ref | Ref | Ref | Ref | Ref | Ref | Ref | Ref | Ref | Ref |
| 0 | 0.30 | -0.68, 1.28 | 0.39 | -0.31, 1.08 | -0.29 | -1.37, 0.81 | **-0.64*** | **-1.22, 0.05** | **0.88*** | **0.19, 1.57** |
| Family structure |  |  |  |  |  |  |  |  |  |  |
| Extended family | Ref | Ref | Ref | Ref | Ref | Ref | Ref | Ref | Ref | Ref |
| Nuclear family | 0.03 | -0.91, 0.97 | -0.12 | -0.82, 0.57 | -0.02 | -1.10, 1.05 | **0.95**** | **0.40, 1.51** | -0.21 | -0.93, 0.52 |
| Family with COVID-19 diagnosis |  |  |  |  |  |  |  |  |  |  |
| No | Ref | Ref | Ref | Ref | Ref | Ref | Ref | Ref | Ref | Ref |
| Yes | 0.10 | -0.15, 1.35 | -0.68 | -1.59, 0.24 | 0.22 | -1.20, 1.64 | 0.22 | -0.63, 1.06 | 0.15 | -0.82, 1.12 |
| Mother’s age | -0.02 | -0.11, 0.08 | -0.01 | -0.07, 0.05 | 0.01 | -0.10, 0.11 | 0.00 | -0.06, 0.06 | -0.01 | -0.08, 0.06 |
| Father’s age | 0.00 | -0.09, 0.09 | 0.03 | -0.03, 0.09 | 0.05 | -0.04, 0.15 | 0.01 | -0.05, 0.06 | -0.02 | -0.09, 0.04 |
| Higher household income before pandemic | -0.18 | -0.56, 0.21 | 0.21 | -0.07, 0.49 | -0.09 | -0.52, 0.35 | 0.04 | -0.21, 0.30 | 0.08 | -0.22, 0.38 |
| Higher household income after pandemic | -0.16 | 0.52, 0.21 | 0.14 | -0.13, 0.41 | -0.10 | -0.52, 0.32 | -0.04 | -0.28, 0.21 | -0.01 | -0.30, 0.28 |
| Length of hospitalization | 0.04 | -0.05, 0.13 | -0.05 | -0.11, 0.02 | 0.07 | -0.03, 0.17 | **0.06*** | **0.00, 0.12** | 0.00 | -0.07, 0.07 |
| Stressful event before pandemic | **0.39**** | **0.15, 0.62** | -0.12 | -0.30, 0.05 | 0.23 | -0.05, 0.51 | 0.12 | -0.04, 0.28 | -0.28** | -0.45, -0.10 |
| Resilience | -0.03 | -0.07, 0.02 | **0.09**** | **0.07, 0.11** | -0.02 | -0.06, 0.03 | -0.02 | -0.05, 0.00 | **-0.02*** | **-0.03, -0.00** |
| Social support | -0.04* | -0.06, -0.01 | **0.03**** | **0.01, 0.05** | -0.02 | -0.05, 0.01 | **-0.02*** | **-0.04, -0.00** | **-0.01*** | **-0.02, -0.00** |
| Caregivers’ PSS scores | 0.03 | -0.03, 0.08 | -0.03 | -0.07, 0.00 | 0.03 | -0.03, 0.09 | 0.00 | -0.03, 0.03 | 0.01 | -0.03, 0.04 |
| Caregivers’ PTGI scores | 0.01 | -0.01, 0.03 | 0.00 | -0.01, 0.01 | -0.00 | -0.02, 0.02 | 0.00 | -0.01, 0.01 | 0.00 | -0.01, 0.02 |
| Caregivers’ GAD-7 scores | **0.18*** | **0.01, 0.34** | -0.03 | -0.15, 0.09 | 0.17 | -0.02, 0.35 | -0.02 | -0.12, 0.09 | -0.03 | -0.15, 0.10 |
| Caregivers’ PHQ-9 scores | **0.17**** | **0.06, 0.28** | **-0.10*** | **-0.17, 0.02** | **0.17**** | **0.05, 0.29** | 0.04 | -0.04, 0.11 | 0.03 | -0.06, 0.11 |
| Caregivers’ PSQI scores | 0.10 | -0.04, 0.25 | **-0.13*** | **-0.22, -0.03** | 0.02 | -0.14, 0.19 | 0.06 | -0.03, 0.15 | 0.03 | -0.08, 0.13 |

COVID-19, coronavirus disease 2019; 95% CI, 95% confidence interval; PTSD, posttraumatic stress disorder; PTGI-SF, Posttraumatic Growth Inventory–Short Form; CDI-S, Children’s Depression Inventory-Short Version; SCARED, Screen for Child Anxiety Related Emotional Disorders; CPSS, Child PTSD Symptom Scale; SDSC, Sleep Disturbance Scale for Children; PSS, PTSD Symptom Scale; PTG-I, Posttraumatic Growth Inventory; PHQ-9, Patient Health Questionnaire, 9-item; GAD-7, Generalized Anxiety Disorder, 7-item; PSQI, Pittsburgh Sleep Quality Index; Ref, reference. **p* < 0.05, ***p* < 0.01.

**eTable 4. Potential risk factors associated with psychiatric symptoms in discharged children in univariate logistic regression model (*n* = 61).**

| **Variable** | **PTSD** | | | **Anxiety** | | | **Depression** | | | **Sleep problem** | | |
| --- | --- | --- | --- | --- | --- | --- | --- | --- | --- | --- | --- | --- |
|  | **OR** | **95% CI** | ***p*** | **OR** | **95% CI** | ***p*** | **OR** | **95% CI** | ***p*** | **OR** | **95% CI** | ***p*** |
| Gender |  |  |  |  |  |  |  |  |  |  |  |  |
| Girl | Ref | Ref | Ref | Ref | Ref | Ref | Ref | Ref | Ref | Ref | Ref | Ref |
| Boy | 0.40 | 0.06, 2.72 | 0.35 | 0.58 | 0.15, 2.32 | 0.44 | 1.22 | 0.35, 4.24 | 0.75 | 0.44 | 0.09, 2.16 | 0.31 |
| Age group |  |  |  |  |  |  |  |  |  |  |  |  |
| 7-12 years old | Ref | Ref | Ref | Ref | Ref | Ref | Ref | Ref | Ref | Ref | Ref | Ref |
| 13-18 years old | 0.55 | 0.06, 5.29 | 0.60 | 0.62 | 0.15, 2.62 | 0.52 | 1.59 | 0.50, 5.05 | 0.43 | 0.33 | 0.04, 3.01 | 0.33 |
| Number of siblings |  |  |  |  |  |  |  |  |  |  |  |  |
| ≥ 1 | Ref | Ref | Ref | Ref | Ref | Ref | Ref | Ref | Ref | Ref | Ref | Ref |
| 0 | 0.71 | 0.11, 4.64 | 0.72 | 0.38 | 0.11, 1.30 | 0.12 | 0.59 | 0.19, 1.79 | 0.35 | NA | NA | NA |
| Family structure |  |  |  |  |  |  |  |  |  |  |  |  |
| Extended family | Ref | Ref | Ref | Ref | Ref | Ref | Ref | Ref | Ref | Ref | Ref | Ref |
| Nuclear family | 1.63 | 0.25, 10.58 | 0.61 | 1.05 | 0.29, 3.77 | 0.94 | **3.99** | **1.29, 12.37** | **0.017** | 1.98 | 0.42, 9.30 | 0.39 |
| Family with COVID-19 diagnosis |  |  |  |  |  |  |  |  |  |  |  |  |
| No | Ref | Ref | Ref | Ref | Ref | Ref | Ref | Ref | Ref | Ref | Ref | Ref |
| Yes | 0.24 | 0.03, 1.73 | 0.16 | 2.51 | 0.28, 22.39 | 0.41 | 0.92 | 0.20, 4.12 | 0.91 | 0.25 | 0.05, 1.33 | 0.10 |
| Mother’s age | 1.07 | 0.91, 1.25 | 0.45 | 0.94 | 0.83, 1.08 | 0.40 | 0.97 | 0.87, 1.07 | 0.51 | 0.95 | 0.80, 1.13 | 0.54 |
| Father’s age | 0.92 | 0.77, 1.10 | 0.35 | 1.02 | 0.92, 1.14 | 0.69 | 0.99 | 0.91, 1.09 | 0.88 | 0.94 | 0.82, 1.08 | 0.40 |
| Higher household income before pandemic | 0.68 | 0.31, 1.52 | 0.35 | 0.88 | 0.52, 1.48 | 0.63 | 1.26 | 0.80, 1.97 | 0.33 | **0.45** | **0.21, 0.96** | **0.040** |
| Higher household income after pandemic | 0.63 | 0.27, 1.48 | 0.28 | 0.94 | 0.57, 1.53 | 0.79 | 1.01 | 0.67, 1.53 | 0.97 | **0.33** | **0.12, 0.95** | **0.039** |
| Length of hospitalization | 1.15 | 0.96, 1.38 | 0.13 | 1.10 | 0.98, 1.24 | 0.12 | **1.13** | **1.00, 1.26** | **0.044** | 0.99 | 0.86, 1.15 | 0.89 |
| Stressful event before pandemic | 1.32 | 0.91, 1.93 | 0.15 | **1.52** | **1.01, 2.28** | **0.044** | 1.08 | 0.81, 1.45 | 0.59 | 0.92 | 0.58, 1.44 | 0.70 |
| Resilience | 0.93 | 0.87, 1.01 | 0.10 | 0.95 | 0.89, 1.01 | 0.10 | 0.95 | 0.90, 1.00 | 0.054 | 0.96 | 0.90, 1.03 | 0.26 |
| Social support | 0.95 | 0.90, 1.00 | 0.053 | **0.96** | **0.92, 1.00** | **0.050** | 0.96 | 0.93, 1.00 | 0.055 | 0.95 | 0.91, 0.99 | 0.032 |
| Caregivers’ PTSD | 3.13 | 0.28, 35.02 | 0.36 | 2.33 | 0.35, 15.61 | 0.38 | 1.81 | 0.28, 11.75 | 0.53 | **12.00** | **1.66, 89.94** | **0.014** |
| Caregivers’ anxiety | 3.92 | 0.56, 27.25 | 0.17 | 1.52 | 0.34, 6.87 | 0.59 | 1.13 | 0.29, 4.41 | 0.86 | 0.58 | 0.07, 5.27 | 0.63 |
| Caregivers’ depression | 2.67 | 0.40, 17.96 | 0.31 | **6.67** | **1.72, 25.82** | **0.006** | 0.63 | 0.18, 2.20 | 0.46 | 3.73 | 0.83, 16.72 | 0.085 |
| Caregivers’ poor sleep quality | 5.57 | 0.58, 53.11 | 0.14 | 0.89 | 0.27, 2.99 | 0.85 | 1.39 | 0.50, 3.87 | 0.54 | 0.97 | 0.23, 4.05 | 0.97 |

PTSD, posttraumatic stress disorder; OR, odds ratio; COVID-19, coronavirus disease 2019; Ref, reference; 95% CI, 95% confidence interval; NA, not available.

**References**

Coffey, S.F., Dansky, B.S., Falsetti, S.A., Saladin, M.E., and Brady, K.T. (1998). Screening for PTSD in a substance abuse sample: psychometric properties of a modified version of the PTSD Symptom Scale Self-Report. Posttraumatic stress disorder. *J Trauma Stress* 11(2)**,** 393-399. doi: 10.1023/a:1024467507565.

Mirzamani, S.M., PhD, MohammadRezaMohammadi, M., Mahmoudi-Gharaei, J., and Mirzamani, M.S. (2007). Validity of The PTSD Symptoms Scale Self Report (PSS-SR) in Iran. *Iranian Journal of Psychiatry* 2(3)**,** 120-123.

Pilz, L.K., Keller, L.K., Lenssen, D., and Roenneberg, T. (2018). Time to rethink sleep quality: PSQI scores reflect sleep quality on workdays. *Sleep* 41(5). doi: 10.1093/sleep/zsy029.

Tedeschi, R.G., and Calhoun, L.G. (1996). The Posttraumatic Growth Inventory: measuring the positive legacy of trauma. *J Trauma Stress* 9(3)**,** 455-471. doi: 10.1007/bf02103658.
